# Low-Density Lipoprotein Cholesterol and Mortality Risk in Elderly Patients Undergoing Valve Replacement Surgery: A Propensity Score Matching Analysis

**DOI:** 10.3389/fnut.2022.842734

**Published:** 2022-04-28

**Authors:** Han-biao Li, Bing-qi Fu, Tong Tan, Xiao-hua Li, Shou-hong Wang, Xue-biao Wei, Zhong-hua Wang

**Affiliations:** ^1^Department of Geriatric Intensive Medicine, Guangdong Provincial Geriatrics Institute, Guangdong Provincial People's Hospital, Guangdong Academy of Medical Sciences, Guangzhou, China; ^2^Department of Cardiology, Guangdong Cardiovascular Institute, Guangdong Provincial Key Laboratory of Coronary Heart Disease Prevention, Guangdong Provincial People's Hospital, Guangdong Academy of Medical Sciences, Guangzhou, China; ^3^Shantou University Medical College, Shantou, China; ^4^Department of Cardiac Surgery, Guangdong Cardiovascular Institute, Guangdong Provincial Key Laboratory of Coronary Heart Disease Prevention, Guangdong Provincial People's Hospital, Guangdong Academy of Medical Sciences, Guangzhou, China

**Keywords:** valve replacement surgery, low-density lipoprotein cholesterol, elderly, prognosis, valvular heart disease (VHD)

## Abstract

**Background:**

The prognostic value of low-density lipoprotein cholesterol (LDL-C) in elderly patients is controversial. This study aimed to elucidate the relationship between the preoperative LDL-C and adverse outcomes in elderly patients undergoing valve replacement surgery (VRS).

**Methods:**

A total of 2,552 aged patients (age ≥ 60 years) undergoing VRS were retrospectively recruited and divided into two groups according to LDL-C level on admission: low LDL-C (<70 mg/dL, *n* = 205) and high LDL-C groups (≥ 70 mg/dL, *n* = 2,347). The association between the preoperative LDL-C with in-hospital and one-year mortality was evaluated by propensity score matching analysis and multivariate analysis.

**Results:**

The mean age was 65 ± 4 years and 1,263 (49.5%) were men. Patients in the low LDL-C group were significantly older (65.9 ± 4.6 vs. 64.9 ± 4.1, *p* = 0.002), with more male (65.4 vs. 48.1%, *p* < 0.001), higher alanine transaminase (ALT) (21 vs. 19, *p* = 0.001), lower serum albumin (35.3 ± 4.6 vs. 37.1 ± 4.1, *p* < 0.001), higher serum creatinine (92.2 ± 38.2 vs.84.6 ± 26.1, *p* = 0.006), lower lymphocyte count (1.7 ± 0.7 vs. 1.9 ± 0.6, *p* < 0.001), lower hemoglobin (121.9 ± 22.3 vs. 130.2 ± 16.5, *p* < 0.001), lower platelet count (171.3 ± 64.3 vs. 187.7 ± 58.7, *p* < 0.001), lower prognostic nutrition index (44 ± 6.2 vs. 46.7 ± 5.8, *p* < 0.001), and more severe tricuspid regurgitation (33.7 vs. 25.1%, *p* = 0.008). The rates of in-hospital death (11.2 vs. 3.7%, *p* < 0.001) and major adverse clinical events (17.6 vs. 9.6%, *p* < 0.001) were significantly higher in the low LDL-C group. The cumulative one-year death rate was significantly higher in the low LDL-C group (Log-Rank = 16.6, *p* < 0.001). After matching analysis and multivariate analysis, no association between LDL-C level and adverse outcomes was detected (all *p* > 0.05).

**Conclusion:**

Our study did not support the negative relationship between LDL-C level and mortality risk in elderly patients undergoing VRS.

## Introduction

Valvular heart disease (VHD) remains to be a major burden worldwide. It was estimated that more than 42 million people worldwide suffered from VHD (1). In developing counties, due to the high incidence of rheumatic heart disease, as well as expanding of the aging population (age ≥ 60 years), hence, with the increase in the incidence of degenerative valvular disease (2, 3), more attention should be drawn to VHD. Valve replacement surgery (VRS) is the treatment of choice for VHD, however, the surgical mortality was high in the elderly population (4, 5). Early identification of these high-risk patients is essential for reducing mortality.

Low-density lipoprotein cholesterol (LDL-C) is recognized as the “bad cholesterol” for its biochemical role in carrying lipids to the periphery and pathogenesis of atherosclerosis (6, 7). Maintaining LDL-C at a lower level is the mainstay of cardiovascular therapeutic principles according to the current guideline, (8, 9) for the benefits of clinical outcome (10–12). However, several studies have demonstrated the role of LDL-C in preventing sepsis and worse clinical outcomes (13, 14) *via* clearing bacterial toxin (13, 15). The prognostic value of LDL-C in elderly patients undergoing VRS remains unclear. This study aimed to elucidate the relationship between the preoperative LDL-C and adverse outcomes in elderly patients undergoing VRS.

## Methods

### Study Population

This study was performed at Guangdong Provincial People's Hospital in China. Consecutive patients aged ≥ 60 years, who received at least one valve replacement, were retrospectively included in the study between January 2010 and December 2017, and were prospectively followed up for one year. Exclusion criteria were: (1) receiving lipid-lowering therapy; and (2) no preoperative LDL-C results.

This study was approved by the Ethics Committee of Guangdong Provincial People's Hospital, with a waiver of informed consent because of the retrospective study design (NO. GDREC2018525H). Oral informed consent was obtained from patients or their relatives by telephone and recorded by trained nurses during the follow-up period.

### Laboratory Investigations and Data Collection

Lipid profiles, including triglycerides and LDL-C, were measured under fasting state in the morning following admission. Ultrasonic cardiograms were performed before surgery and left ventricular function was evaluated using the biplane Simpson's method. The estimated glomerular filtration rate (eGFR) was calculated using the equation of Chronic Kidney Disease Epidemiology Collaboration (CKD-EPI) (16). Prognostic nutrition index (PNI) was calculated using the following formula: [10 × albumin (g/dl)] + [0.005 × absolute preoperative lymphocyte count (/mm^3^)] (17). Clinical data were collected using an electronic case report form, and were obtained and randomly confirmed by two independent researchers.

### Follow-Up and Clinical Outcomes

Clinical follow-up was carried out by telephone interview, inquiry of readmission clinical records, and outpatient clinic interviews. The primary study endpoint was defined as in-hospital mortality. Postoperative one-year death and in-hospital major adverse clinical events (MACEs; including acute heart failure, dialysis, stroke, or death) were considered as the secondary endpoints.

### Statistical Analysis

Continuous variables are expressed as mean ± standard deviation (SD) or median (first and third quartile) based on data distribution. Independent sample *t* or non-parametric tests were conducted accordingly. Categorical variables are presented as numbers and percentages and were compared using chi-squared tests. Cumulative survival curves were generated using the Kaplan-Meier method and data survival were compared among groups using log-rank tests. We conducted a 1:1 propensity scores matching analysis (PS analysis) using the nearest-neighbor method, with a.01 caliper size to reduce selection bias and confounding factors. Variables that differed significantly between groups, including age, gender, ALT, serum albumin, creatinine, lymphocyte count, hemoglobin, platelet, PNI, tricuspid regurgitations, were involved in the PS analysis. The linear correlation between two variables was analyzed by the Pearson correlation coefficient. Multivariate logistic regression and Cox survival analyses were also conducted to determine the independent contribution of LDL-C to clinical outcomes. All data were analyzed using SPSS software version 22.0 (SPSS, Inc., Chicago, Illinois), and *p* < 0.05 was considered statistically significant.

## Results

### Baseline Characteristics

**Figure 1:**
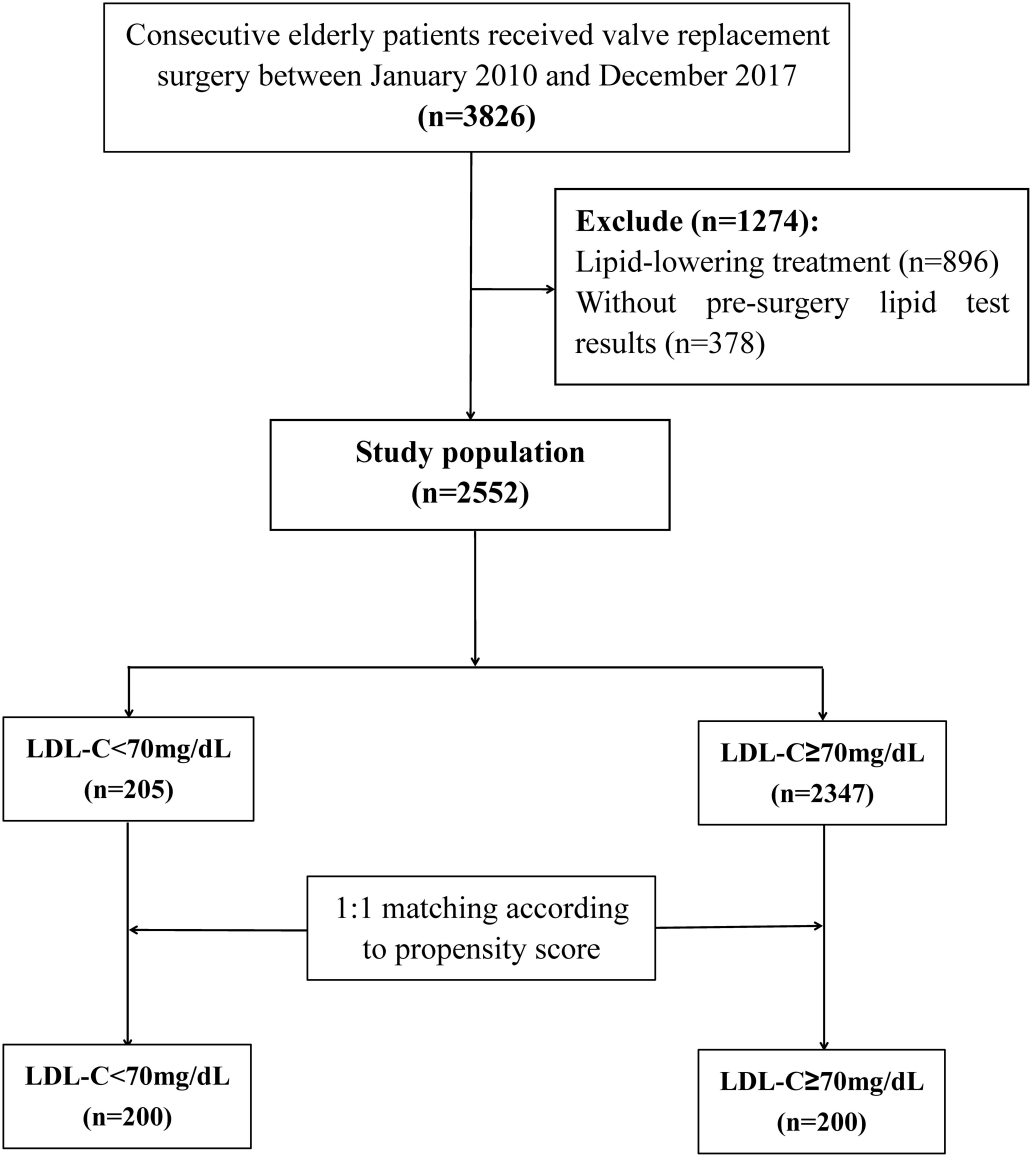
Flow chart of the study sample with inclusion and exclusion from the analysis.

A total of 3,826 patients undergoing VRS were screened, 896 received lipid-lowering treatment, and 378 without pre-surgery lipid test results were excluded. At last, 2,552 individuals were included in the statistical analysis (Figure 1). Among these patients, 1,263 (49.5%) were men and 1,289 (50.5%) were women, and the mean age was 65 ± 4 years. The in-hospital mortality was 4.3%. According to previous study (18), the included patients were classified into two groups: <70 mg/dL (*n* = 205) and ≥ 70 mg/dL (*n* = 2347). Baseline characteristics for the study population were presented in Table 1.

**Table 1:** Baseline clinical characteristics.

**Variables**	**All patients**		***P*-value**	**Propensity-Matched patients**		***P*-value**
	**LDL-C <70 mg/dL (*n =* 205)**	**LDL-C ≥70 mg/dL (*n =* 2347)**		**LDL-C <70 mg/dL (*n =* 200)**	**LDL-C ≥70 mg/dL (*n =* 200)**	
Age (year)	65.9 ± 4.6	64.9 ± 4.1	0.002	65.9 ± 4.4	66.2 ± 5.0	0.547
Sex, *n* (%)						
Male	134 (65.4)	1129 (48.1)	<0.001	130 (65.0)	119 (59.5)	0.257
Female	71 (34.6)	1218 (51.9)		70 (35.0)	81 (40.5)	
Hypertension, *n* (%)	65 (31.7)	637 (27.1)	0.160	64 (32.0)	55 (27.5)	0.325
Diabetes, *n* (%)	26 (12.7)	222 (9.5)	0.135	25 (12.5)	17 (8.5)	0.192
NYHA III-IV, *n* (%)	73 (35.6)	790 (33.7)	0.571	71 (35.5)	76 (38.0)	0.604
ALT (U/L)	21.0 (16.0, 29.6)	19.0 (15.0, 26.0)	0.001	21.0 (16.0, 29.0)	19.0 (14.0, 28.8)	0.110
Serum albumin (g/L)	35.3 ± 4.6	37.1 ± 4.1	<0.001	35.4 ± 4.5	35.7 ± 4.7	0.513
Serum creatinine (umol/L)	92.2 ± 38.2	84.6 ± 26.1	0.006	91.4 ± 36.4	87.4 ± 26.4	0.212
eGFR (mL/min/1.73 m^2^)	73.2 ± 19.7	75.5 ± 17.3	0.106	73.4 ± 19.3	74.3 ± 18.5	0.643
WBC count (10^9^/L)	6.7 ± 2.3	6.8 ± 2.0	0.446	6.6 ± 2.2	6.7 ± 1.9	0.686
Neutrophil count (10^9^/L)	4.1 ± 2.0	4.1 ± 1.8	0.806	4.0 ± 1.8	4.2 ± 1.8	0.434
Lymphocyte count (10^9^/L)	1.7 ± 0.7	1.9 ± 0.6	<0.001	1.7 ± 0.7	1.7 ± 0.6	0.492
Hemoglobin (g/L)	121.9 ± 22.3	130.2 ± 16.5	<0.001	121.8 ± 22.4	120.7 ± 18.7	0.589
PLT (10^9^/L)	171.3 ± 64.3	187.7 ± 58.7	<0.001	172.3 ± 64.7	170.8 ± 52.2	0.795
PNI	44.0 ± 6.2	46.7 ± 5.8	<0.001	44.1 ± 6.1	44.2 ± 6.0	0.891
CRP (mg/L)	3.2 (1.8,7.7)	2.9 (1.8,5.6)	0.122	3.2 (1.7,7.7)	2.8 (1.8,6.4)	0.409
Severe valve disease, *n* (%)						
Aortic stenosis	35 (17.1)	170 (82.9)	0.165	35 (17.5)	41 (20.5)	0.444
Aortic regurgitation	52 (25.4)	532 (22.7)	0.378	50 (25.0)	47 (23.5)	0.726
Mitral stenosis	35 (17.1)	540 (23.0)	0.051	34 (17.0)	38 (19.0)	0.603
Mitral regurgitation	87 (42.4)	936 (39.9)	0.474	84 (42.0)	85 (42.5)	0.919
Tricuspid regurgitation	69 (33.7)	590 (25.1)	0.008	68 (34.0)	56 (28.0)	0.195
LVEF (%)	61.7 ± 9.6	61.5 ± 9.8	0.744	61.9 ± 9.5	62.4 ± 10.2	0.571
Type of surgery, *n* (%)						
AVR	115 (56.1)	1341 (57.1)	0.773	113 (56.5)	106 (53.0)	0.482
MVR	143 (69.8)	1641 (69.9)	0.961	139 (69.5)	143 (71.5)	0.661
TVI	131 (63.9)	1410 (60.1)	0.283	127 (63.5)	128 (64.0)	0.917
CABG	13 (6.3)	149 (6.3)	0.997	13 (6.5)	17 (8.5)	0.448

*LDL-C, low-density lipoprotein cholesterol; NYHA, New York Heart Association; ALT, alanine transaminase; eGFR, estimated glomerular filtration rate; WBC, white blood cell; PLT, platelet; PNI, prognostic nutrition index; CRP, C-reactive protein; LVEF, left ventricular ejection fraction; AVR, aortic valve replacement; MVR, mitral valve replacement; TVI, tricuspid valve intervention; CABG, coronary artery bypass grafting*.

We found that the patients in the group with lower LDL-C were significantly older (65.9 ± 4.6 vs. 64.9 ± 4.1, *p* = 0.002) and more likely to be male (65.4 vs. 48.1%, *p* < 0.001) than the group with higher LDL-C. Additionally, the proportion of patients with higher ALT (21 vs. 19, p = 0.001), lower serum albumin (35.3 ± 4.6 vs. 37.1 ± 4.1, *p* < 0.001), higher serum creatinine (92.2 ± 38.2 vs. 84.6 ± 26.1, p = 0.006), lower lymphocyte count (1.7 ± 0.7 vs. 1.9 ± 0.6, *p* < 0.001), lower hemoglobin (121.9 ± 22.3 vs. 130.2 ± 16.5, *p* < 0.001), lower platelet count (171.3 ± 64.3 vs. 187.7 ± 58.7, *p* < 0.001), lower PNI (44 ± 6.2 vs. 46.7 ± 5.8, *p* < 0.001), and severe tricuspid regurgitation (33.7 vs. 25.1%, p = 0.008) were significantly higher in the lower LDL-C group.

### LDL-C and Clinical Outcomes

**Figure 2:**
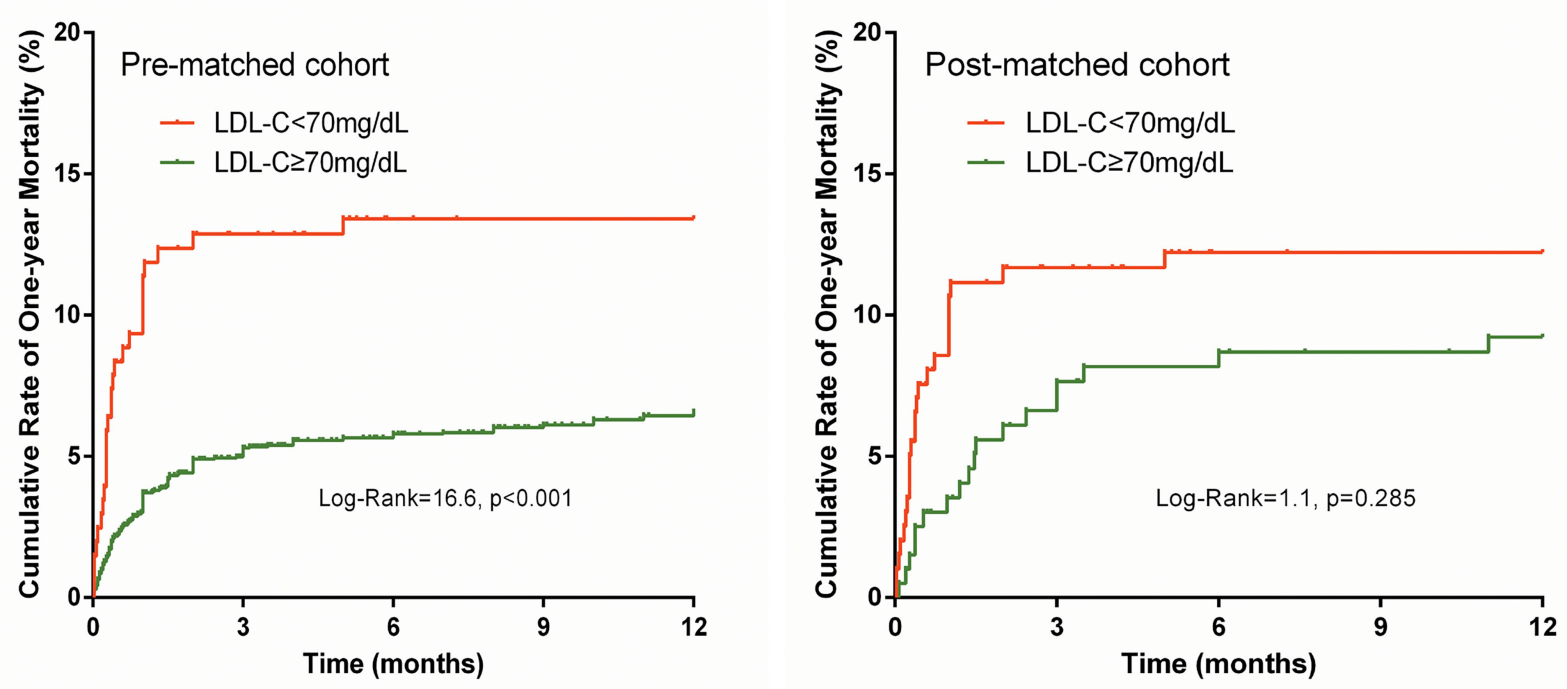
Cumulative incidence curves for one-year mortality.

**Figure 3:**
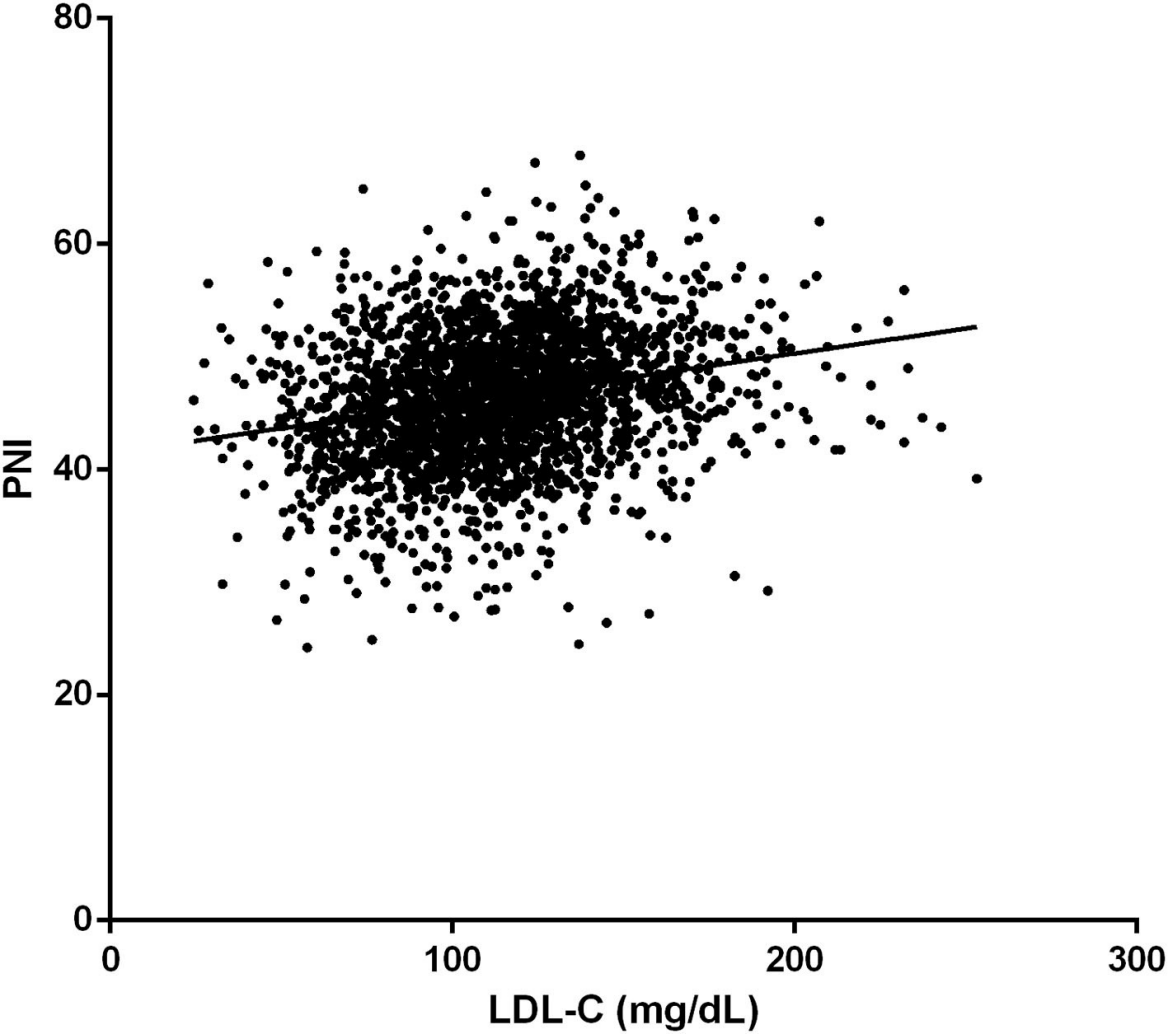
The correlation of low-density lipoprotein cholesterol (LDL-C) and Prognostic Nutrition Index (PNI).

The prevalence of in-hospital adverse events was presented in Table 2. Collectively, during hospitalization, 110 (4.3%) patients died, and 261 (10.2%) patients suffered from MACEs. The in-hospital mortality (11.2 vs. 3.7%, *p* < 0.001) and MACEs (17.6 vs. 9.6%, *p* < 0.001) were higher in patients with lower LDL-C. Among patients who completed one-year follow-up after surgery (n = 2062; 92.8%), the one-year mortality was also significantly higher in the group with lower LDL-C (Log-Rank = 16.6, *p* < 0.001; Figure 2). The LDL and PNI were positively correlated and the coefficient of Pearson's correlation was.241 (*p* < 0.001, Figure 3).

**Table 2:** Association between LDL-C and clinical outcomes.

	**LDL-C <70 mg/dL**	**LDL-C ≥70 mg/dL**	**P-value**
**Pre-matched cohort**	n = 205	n = 2,347	
In-hospital death, n (%)	23 (11.2)	87 (3.7)	<0.001
In-hospital MACEs, n (%)	36 (17.6)	225 (9.6)	<0.001
**Post-matched cohort**	n = 200	n = 200	
In-hospital death, n (%)	20 (10.0)	13 (6.5)	0.203
In-hospital MACEs, n (%)	33 (16.5)	24 (12.0)	0.198

*LDL-C, low-density lipoprotein cholesterol; MACEs, major adverse clinical events*.

### Propensity Scores Matching Analysis

The PS analysis generated 200 patients with LDL-C < 70 mg/dL and 200 with LDL-C ≥ 70mg/dL. After matching, the baseline clinical characteristics were well-balanced between the two groups (Table 1). The lower LDL-C group did not show any statistically significant results of higher or lower risk of in-hospital mortality, MACEs, or one-year morality compared to the higher LDL-C group (all p > 0.05, Table 2 and Figure 2).

### Multivariate Analysis

After adjustment for potential confounding factors, LDL-C was not an independent risk factor for in-hospital death [odds ratio (OR) = 0.77, 95% confidence interval (95% CI):.57–1.03, p = 0.077; Table 3], in-hospital MACEs (OR =.95, 95% CI:.79–1.14, p = 0.560; Table 3), and one-year mortality [hazard ratio (HR) = 1.08, 95% CI:.88–1.32, p = 0.480; Table 3].

**Table 3:** Unadjusted and adjusted OR/HR of LDL-C.

	**OR/HR**	**95% CI**	***P***
**In-hospital death**			
Model 1: unadjusted	0.67	0.53, 0.86	0.001
Model 2: multivariate adjusted ^a^	0.77	0.57, 1.03	0.077
**In-hospital MACEs**			
Model 1: unadjusted	0.80	0.68, 0.94	0.006
Model 2: multivariate adjusted ^b^	0.95	0.79, 1.14	0.560
**One-year mortality**			
Model 1: unadjusted	0.81	0.67, 0.97	0.022
Model 2: multivariate adjusted ^c^	1.08	0.88, 1.32	0.480

a *Adjusted for age, NYHA III-IV, ALT, eGFR < 60 mL/min/1.73 m^2^, anemia, PLT, PNI, CRP, severe tricuspid regurgitation, LVEF, CABG*.

b *Adjusted for age, diabetes, NYHA III-IV, eGFR < 60 mL/min/1.73 m^2^, neutrophil count, anemia, PLT, PNI, CRP, severe tricuspid regurgitation, LVEF, CABG*.

c *Adjusted for age, NYHA III-IV, ALT, eGFR < 60 mL/min/1.73 m^2^, WBC, neutrophil count, anemia, PLT, PNI, CRP, severe tricuspid regurgitation, LVEF, mitral valve replacement, tricuspid valve intervention, CABG*.

*LDL-C, low-density lipoprotein cholesterol; OR, odd ratio; HR, hazard ratio; CI, confidence interval; MACEs, major adverse clinical events; NYHA, New York Heart Association; ALT, alanine transaminase; eGFR, estimated glomerular filtration rate; PLT, platelet; PNI, prognostic nutrition index; CRP, C-reactive protein; LVEF, left ventricular ejection fraction; CABG, coronary artery bypass grafting*.

## Discussion

In this study, we explore the relationship between the preoperative LDL-C level and the rate of postoperative adverse events in elderly patients (≥ 60 years old) undergoing VRS. The univariate analysis showed that lower LDL-C level was associated with a higher risk of in-hospital and one-year mortality. However, this association disappeared after PS analysis and multivariate analysis, where potential confounding factors were matched or adjusted. Therefore, the negative relationship between LDL-C level and the clinical outcome might be confounded by clinical characteristics in the lower LDL-C group.

On one hand, as a carrier of cholesterol, the LDL-C has been well-accepted as a critical risk factor for developing atherosclerosis (19). Besides, elevated LDL-C was linearly correlated with the risk of unfavorable events (20), and the reduced amount of LDL-C was associated with a corresponding reduction in the risk of cardiovascular and cerebrovascular events (21, 22); consequently, lipid-lowering drugs, such as statins, were recommended by both European Society of Cardiology (ESC) and American Heart Association (AHA) guidelines, indicating that LDL-C level should be as low as possible (8, 9).

On the other hand, LDL-C exhibited a protective effect in infectious diseases through its function of delivering toxic bacterial productions for hepatic elimination (23), thus patients with lower LDL-C levels were more likely to develop sepsis (13–15). Based on this theory, several studies have proved the link between lower LDL-C levels and higher mortality afterward, especially for elderly patients (24, 25). A similar result was found in our pre-matched analysis. However, no association between LDL-C level and mortality risk was found after matching the confounding factors. Although the exact mechanism is unclear, the following aspects from previous studies might account for our findings.

Firstly, the patients in the group with lower LDL-C were significantly older in our study. Aging is a complicated physiological process, where the function of all organ systems fades away (26). The antioxidative capacity declines with age, resulting in excessive oxidative stress in the cells (27). Reactive oxidative species (ROS) alters cholesterol metabolism by decreasing the acetyl-CoA acetyltransferase (ACAT) activity and the secretion rate of very low-density lipoprotein cholesterol, which could explain the decrease in LDL-C level as aging (28). Besides, older age was an independent risk factor for worse clinical outcomes. After matching the age and other variables between groups, LDL-C level was not associated with clinical outcomes in elderly patients undergoing VRS, which implied that LDL-C may not be able to influence the outcome independently and individual baseline characteristics should be considered as a whole during clinical evaluation.

Secondly, a significant positive correlation between PNI and LDL-C levels was found in our study. The PNI is a frequently used index in clinical practice to assess one's nutritional status and has been proven to be positively correlated with prognosis in multiple situations, such as surgical high-risk and elderly patients (29). As for VRS, a similar correlation was verified by Cho et al. (30) and Gürbak et al. (31). Thus, the significant relationship between LDL-C and mortality before matching in our study may be the result of the confounding effect of PNI.

In a summary, both older age and worse nutritional status were related to lower LDL-C levels, hence, could be the confounding factors in associating LDL-C level with clinical outcome after VRS. During clinical evaluation, LDL-C level should not be used independently to predict clinical outcome, instead, other clinically significant factors such as age and nutritional status should be integrated.

## Limitations

Yet, our current study had several limitations. First, due to the shortcomings inherent in the retrospective design, the underlying mechanism of low LDL-C level on admission was yet unknown; however, the primary aim of this study was to investigate the prognostic value of LDL-C in patients undergoing VRS. Second, the sample size was relatively small in the propensity score matching analysis. Finally, LDL-C was obtained once on admission, whether the change of LDL-C level during hospitalization would affect the clinical outcomes cannot be drawn from our findings.

## Conclusion

The present study was the first to investigate the prognostic value of LDL-C level in elderly patients undergoing VRS. Low LDL-C level was associated with increased risk of in-hospital and one-year mortality. However, after PS analysis and multivariate analysis, the association disappeared. The prognostic effect of LDL-C needs to be further evaluated and confirmed by large sample, prospective, and randomized controlled studies.

## Data Availability Statement

The raw data supporting the conclusions of this article will be made available by the authors, without undue reservation.

## Ethics Statement

The studies involving human participants were reviewed and approved by the Ethics Committee of Guangdong Provincial People's Hospital. The Ethics Committee waived the requirement of written informed consent for participation.

## Author Contributions

S-hW, X-bW, and Z-hW were involved in the conception and design of this study. H-bL, B-qF, TT, and X-hL contributed to the data collection and statistical analysis. H-bL and B-qF constructed the manuscript, which was revised and approved by all the authors for publication. All authors contributed to the article and approved the submitted version.

## Funding

This study was supported by grants from the National Natural Science Foundation of China (Grant No. 82002014), Natural Science Foundation of Guangdong Province (Grant No. 2021A1515010107), Science and Technology Projects of Guangzhou (Grant No. 201903010097), and Guangdong Provincial Key Laboratory of Coronary Heart Disease Prevention (Grant No. 2017B030314041). The funders had no role in the study design, data collection, analysis, decision to publish, or preparation of the manuscript.

## Conflict of Interest

The authors declare that the research was conducted in the absence of any commercial or financial relationships that could be construed as a potential conflict of interest.

## Publisher's Note

All claims expressed in this article are solely those of the authors and do not necessarily represent those of their affiliated organizations, or those of the publisher, the editors and the reviewers. Any product that may be evaluated in this article, or claim that may be made by its manufacturer, is not guaranteed or endorsed by the publisher.

## Abbreviations

VHD: valvular heart disease
LDL-C: low-density lipoprotein cholesterol
VRS: valve replacement surgery
CHD: coronary heart disease
eGFR: estimated glomerular filtration rate
CKD-EPI: Chronic Kidney Disease Epidemiology Collaboration
MACEs: major adverse clinical events
NYHA: New York Heart Association
ALT: alanine transaminase
WBC: white blood cell
PLT: platelet
CRP: C-reactive protein
PNI: prognostic nutrition index
LVEF: left ventricular ejection fraction
AVR: aortic valve replacement
MVR: mitral valve replacement
TVI: tricuspid valve intervention
CABG: coronary artery bypass grafting.
